# The assessment of *Oreochromis mossambicus* muscle tissue and the yield performance of *Solanum tuberosum* in a small-scale sandponics system

**DOI:** 10.1007/s11356-025-36495-0

**Published:** 2025-05-14

**Authors:** Refilwe Lukhwareni, Philiswa Nosizo Nomngongo, Uwineza Marie Clementine Nibamureke, Kgaohelo Moila, Nomhlekhabo Wendy Sekete, Godfrey Tshokolo Ndamane, Henry Akum Njom, Lucky Sithole, Michael Rudolph, Nomali Ziphorah Ngobese

**Affiliations:** 1 Department of Zoology, University of Johannesburg, PO BOX 524, Johannesburg, 2006 South Africa; 2 Department of Chemical Sciences, Faculty of Science, University of Johannesburg, PO BOX 524, Johannesburg, 2006 South Africa; 3 Department of Biological Sciences, Faculty of Science, Engineering & Agriculture, University of Venda, Private Bag X5050, Thohoyandou, 0950 South Africa; 4 Centre for Ecological Intelligence, Department of Electrical and Electronic Engineering, Faculty of Engineering and Build Environment, University of Johannesburg, Johannesburg, South Africa; 5 Agricultural Research Council, Private Bag X1251, Potchefstroom, 2531 South Africa; 6 Department of Agriculture and Rural Development, Pietermaritzburg, 3245 South Africa; 7 Unit for Environmental Sciences and Management, Faculty of Natural and Agricultural Sciences, North-West University, Private Bag X6001, Potchefstroom, South Africa

**Keywords:** Organic agriculture, *Oreochromis mossambicus*, Sandponics, Skeletal muscle histology, Water quality, Potatoes

## Abstract

Aquaponics, integrating hydroponics and aquaculture in a circular system, offers a promising approach to addressing food and nutrition security while promoting water conservation in South Africa. This technology is a sustainable means of food production that minimizes environmental waste by simultaneously cultivating plants and rearing fish. This study aimed to evaluate the histology of muscle tissue in Mozambique tilapia (*Oreochromis mossambicus*) and the performance of Irish potato (*Solanum tuberosum*) in a small-scale sandponics system. Two potato cultivars (Moonlight and Taurus) were planted in a system linked to a 1000-L water tank containing 25 sexually mature Mozambique tilapia from January to June 2023. Fish histology and potato yield performance were assessed to gauge the efficiency of the system and to generate baseline data for future studies. Results showed that tuber production in the sandponics system was comparable to field conditions, with the Moonlight cultivar yielding the heaviest tubers (293–307 g per plant) with a short-oval shape, demonstrating its superior adaptability to this system. Taurus yielded lighter tubers (139–168 g per plant) that were either round or short oval depending on the grow beds used for production. Fish histological analysis revealed a higher prevalence of muscle tissue alterations in the control group compared to the experimental group. However, both groups displayed a similar condition factor (*p* < 0.05), indicating good overall health. Despite the promising results, the significantly high levels (*p* < 0.05) of metal accumulation (As, Cu, Mn, and Zn) in the fish were observed, raising concerns about their suitability for human consumption. This study demonstrates that sandponics systems can effectively support potato production with fish maintaining good general health. However, further investigation is needed to mitigate metal accumulation to ensure the safety of fish for consumption.

## Introduction

According to the Food and Agriculture Organization (2022), global food production must increase by 50% as the human population continuously increases in urban settlements to meet food security targets (Obirikorang et al. 2021). With such a huge increase, the need to secure food in limited space has become one of the growing concerns in South Africa. This is because of the limited water resources in a water-scarce country and land crop production, especially in urban and periurban settings (Meza et al. 2021). Therefore, society must look at alternative food-growing systems like hydroponics, a branch of urban farming that can address the high demand for food production in limited spaces (Wirza and Nazir 2021; Somerville et al. 2014). Hydroponics is used to grow plants in a nutrient-rich water medium (soil-less agriculture) that affords farmers precise control and delivery of nutrients and environmental conditions (Rajaseger et al. 2023). This makes hydroponics distinctly beneficial compared with conventional open-field farming as the latter leaves crops vulnerable to several biotic and abiotic threats that can compromise yields (e.g., diseases and detrimental weather conditions associated with climate change). The most notable differences between open-field and hydroponics are that the latter allows for the efficient use of space and is effective at conserving most of the water used in crop production (Pomoni et al. 2023; Rajaseger et al. 2023).

Aquaponics, on the other hand, represents an advanced application of technology that has been studied to enhance efficiency in sustainable agriculture (Sewilam et al. 2022; Baganz et al. 2021; Goddek et al. 2015). The technology is a bio-integrated system linking hydroponics (soil-free crop production in a nutrient solution) with aquaculture (fish farming), which presents an environmentally friendly alternative for producing both the animal protein and vegetables required for a balanced diet (Krastanova et al. 2022; Obirikorang et al. 2021). Sandponics is an alternative and unique cultivation system developed in the 1970 s using a soil-free greenhouse facility. The system has shown the advantages of the high quality of products, simple water supply management, and reduced facility costs. It is expected to be a promising cultivation system for producing high-quality vegetables (Sewilam et al. 2022; Baba and Ikeguchi 2015). These systems operate through a natural nitrogen cycle that allows fish effluent to be recirculated and act as a nutrient source to support plant growth and development: (1) nitrogen waste is released as ammonia through a process called ammonification and this ammonia is converted to nitrite by *Nitrosomonas* bacteria in the water medium; (2) bacteria from the *Nitrobacteraceae* family turn the nitrite into nitrate through nitrification; then (3) plants use this nitrate as their source of nutrients. This reduces the need for synthetic nitrogen fertilizers and saves up to 90% of the water used for irrigation in open-field crop production (Tyson et al. 2011). Kok et al. (2024) reported that maintaining optimal water quality is crucial for the health of both fish and plants which requires continuous monitoring and management, and which can be technically demanding. Additionally, in areas where water quality is poor, hydroponic systems can integrate water treatment technologies to make use of greywater/brackish or saline water that would otherwise be unsuitable for traditional agriculture (Ibrahim et al. 2023; Magwaza et al. 2020; Maucieri et al. 2018). There are several hydroponic designs available in the market, and these include bench beds, nutrient film techniques, floating rafts, and different types of media such as rock wool, perlite, and pine bark (Tyson et al. 2011). The crucial role of microbial communities in nutrient cycling and system efficiency was highlighted by Kushwaha et al. (2023).

However, limited studies have shown a net benefit of aquaponics and its impact on the quality of crops produced and the health of the fish used during crop production. Of note is the study by Rakocy et al. (2004), who reproduced the *Oreochromis* species for 4 years in a commercial scale aquaponic system and evaluated the performance of basil and three okra varieties. They observed faster growth and higher yields compared to field production. Yep and Zheng (2019), in their review, found that *Oreochromis mossambicus* or *niloticus* fish and dark leafy vegetables are commonly paired in aquaponics studies. They suggested that growing high-value flowering crops like sweet peppers, tomatoes, or cucumbers might be challenging due to not having the right nutrient balance in an aquaponic system, as the water medium tends to have low potassium (K^+^), magnesium (Mg^+^), and calcium (Ca^+)^ levels. However, Krastanova et al. (2022) confirmed the popularity of *Oreochromis* species and basil as the most popular crop in such systems, followed by lettuce and tomato. Both studies pointed out that future research should investigate the biological aspects of such systems and how certain microbes can help plants absorb nutrients better. The popularity of these species is linked to histopathology as the fish is a rapid and valuable biomarker in assessing the effects of toxicants on the health of target organs such as muscles (Krastanova et al. 2022; Sawsan et al. 2017; Van Dyk et al. 2012). Monitoring changes in microbial species and the bioaccumulation of minerals and trace elements is essential for ensuring the food safety of both fish and crops produced (Jooste et al. 2014). When the levels of metals and trace metals are above the food safety standards guidelines, it could lead to adverse health effects for consumers.

Therefore, the present study focuses on assessing the performance of *O. mossambicus* and potatoes in a small-scale sandponics system and monitoring changes in water physicochemical and microbial quality, as a way to understand the benefits derived from such a system.

## Materials and methods

### Experimental setup

**Fig. 1:**
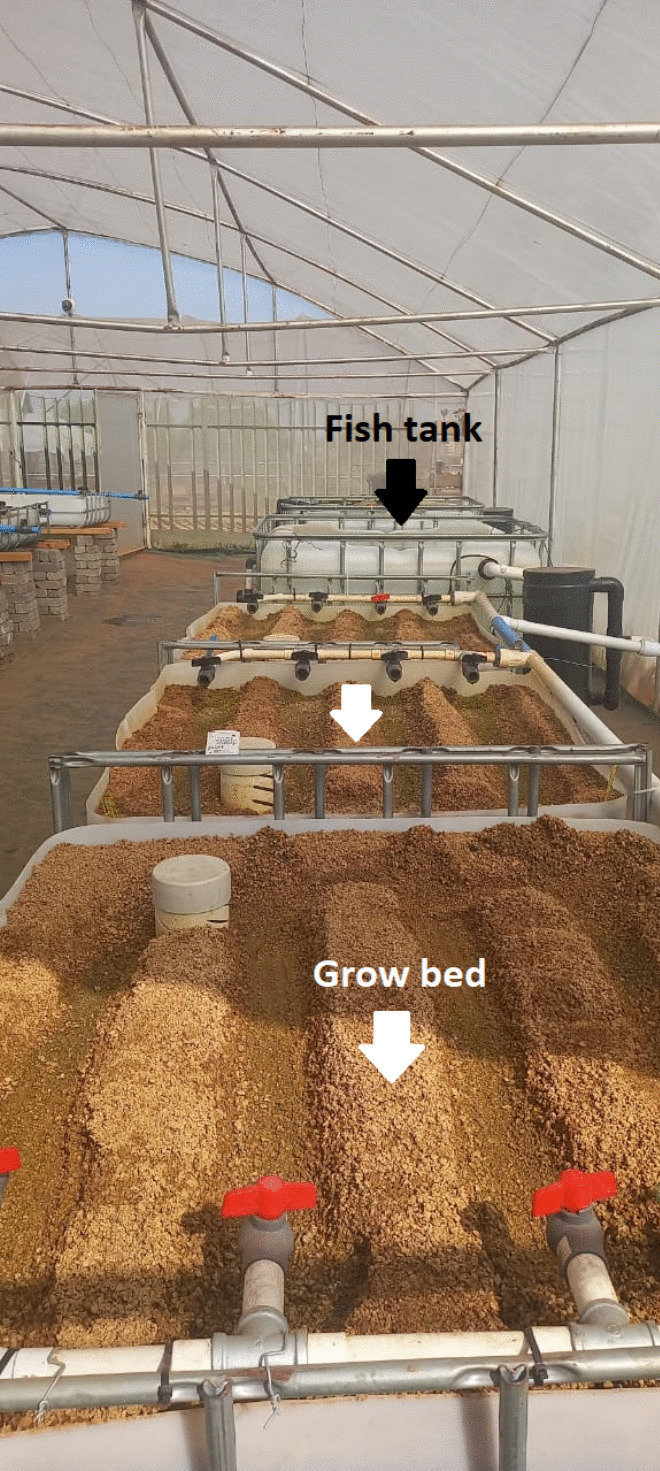
The experimental setup: comprised of one fish tank (1000 L) connected to three sand-filled grow beds

This project was approved by the Faculty of Science Ethics Committee of the University of Johannesburg (UJ) (reference no: 2023-04−11/Lukhwareni) and the experiment was conducted in January–June 2023. Two small-scale closed-loop sandponics systems were assembled. These were composed of three grow beds filled with a river sand medium connected to a fish tank (Figure 1). The tanks were attached to the grow beds through a biological filter and contained 25 *Oreochromis mossambicus* housed in a shade net tunnel. A similar setup was tested by Salam et al. (2014), where they compared the performance of tomatoes grown in sawdust, gravel, or bricklet media revealing gravel as the favourable medium.

One system was used as the experimental system, and another was used as a control (no potatoes were planted). The fish were laboratory-bred and were quarantined in a research aquarium for more than a year before the experiment commenced. The system functioned as follows: wastewater was pumped from the fish tank directly to the grow beds for irrigation using a 28-W submersible pump for the control system and a 0.37 kW peripheral pump for the experimental tank. The water in the fish tank was aerated with a 60-W air pump to supply oxygen. The irrigated water passed through the sand and potato tubers/plants to supply nutrients. The water from the grow beds was filtered through an auto-bell siphon—a standpipe, bell pipe, and a gravel guard fitted with plastic mesh and passed through a sump tank before returning to the fish tank. The plants and fish were monitored over the crop growth period and changes observed were recorded.

### Potato planting procedures and growth performance analyses

Two potato cultivars, Taurus (Wesgrow Potatoes, South Africa) and Moonlight (Potato Seed Production), were sown in two grow beds during the potato production season. Both cultivars are high-yielding, early-medium maturing and are white-fleshed potatoes (Anderson et al. 2004; Siano et al. 2024). The former is a multipurpose cultivar, while Taurus is a crisping cultivar. Both were tested in the same grow beds to test the robustness of the system in supporting genetic diversity. Over this growth season, late blight was observed, and a 20% apple cider vinegar solution was administered as a foliar spray to mitigate the infection. Potato aphids were observed on the leaves and an organic pesticide (Pyrol, 200 mL in 10 L of water) was sprayed as a deterrent. Water samples were collected for microbial analysis upon observation of leaf browning associated with blight developments. Potatoes were harvested manually 2 weeks following observation of shoot dieback (senescence) and placed in Ziploc bags. All harvested potatoes were weighed to determine the total yield (t/ha), with no physical defects present, and manually sorted according to commercial size classification standards (i.e., large [> 250 g], medium [100–250 g], or small and baby [< 100 g]) to establish the size frequency distribution (Ngobese et al. 2022). Digital Vernier Calipers were used to determine the length and width of each tuber, and these were used to calculate the tuber form index using the following equation 1 below. The tuber form index was used to categorize shapes as either round (< 109), short-oval (110–129), oval (130–149), long-oval (150–1.69), or long (≥ 170).

1 $$\text{TFI }=\text{ Length }/\text{ Width}$$

Nutritional analysis followed the methodology outlined by Ngobese et al. (2017). Moisture content was assessed using a Kern MLB 50-3 N moisture meter (Germany). Fiber analysis calculated the acid detergent fiber (ADF) and neutral detergent fiber (NDF) fractions. Samples were digested in respective acid-detergent and neutral-detergent solutions using a digestion block. The Somogyi-Nelson assay and spectrophotometry methods were employed for quantifying reducing sugars and starch. Fat content was measured through ether extraction. For mineral analysis (Na, P, Ca, Mg, K, Cu, Fe, Zn, Mn, N), the remaining samples were prepared using microwave-assisted acid digestion. Approximately 500 mg of the sample was combined with 10 mL of 65% nitric acid in a vessel. These vessels, containing both the sample and standard solutions, were placed in a MARS 6240/50 microwave system (Matthews, NC, USA). The digestion process involved a plant material program with a ramp time of 20–25 min at 200 °C, followed by a 10-min hold. After digestion, the vessel pressure was released, and the solution was transferred to 10-mL Falcon tubes. The mineral content was analyzed using ICP-OES under the following conditions: 1.3 kW radiofrequency power, 150 kPa nebulizer pressure, 15 L/min plasma argon flow, and 1.5 L/min auxiliary argon flow. The protein content was estimated by multiplying the total nitrogen content, obtained from ICP-OES, by a conversion factor of 6.25 to calculate crude protein. Total carbohydrate content was derived from the combined sugar, fiber, and starch measurements.

### Exposure of fish and fish health analyses

Fifty sexually mature *O. mossambicus* fish were quarantined at the UJ Research Aquarium for a year before being translocated to the shade net tunnel at the Centre for Ecological Intelligence’s (CEI) research hub on the Bunting Road Campus. At the center, fish were acclimated in 1000-L plastic tanks attached to a biological filter for 6 weeks before the potato tubers were planted. On the planting day, 25 fish were moved to the experimental tank (cultivated tubers), and the other half was left in the control tank which served as an uncultivated group. Fish were fed Aqualapia pellets midmorning daily, and tanks were cleaned weekly for the duration of the experiment. After the exposure period, 10 days before harvesting the potatoes, fish were sampled from the tanks and weighed (g) and the total length (mm) was recorded to calculate Fulton’s condition factor (CF) according to Carlander (1969). The CF was calculated (CF = Weight (g)/Length^3^ (mm) × 100) to quantify the general health condition of the fish following exposure. Fish were ethically killed by severing the spinal cord after being sedated with a damp cloth (Van Dyk et al. 2009).

Each fish was then macroscopically assessed to identify any external abnormalities, parasites, or injuries. The skeletal muscle fillet was sampled on the dorsal part of the fish and fixed in 10% neutral buffered formalin for further histological analysis following standard techniques for hematoxylin and eosin (H&E) staining. All tissues were analyzed qualitatively on a multi-head light microscope (Olympus, Wirsam) by two assessors, and micrographs were taken with a Zeis microscope with Axioplan Imaging software. Each of the identified histological alterations’ percentage prevalence was calculated. For chemical analysis, 1 g of muscle was sampled and placed in zip bags and then stored at −20 °C until further analysis.

### Water physicochemical properties

In situ water quality parameters, including temperature (°C), pH, total dissolved salts (ppm), dissolved oxygen (%), and electrical conductivity (µS/cm), were recorded weekly using Eutech handheld water quality meters. For ex situ analyses, 500 mL water samples were collected in plastic bottles before planting and post-harvest, kept at 4 °C, and later analyzed for nitrates (mg/L), nitrite (mg/L), total phosphates (mg/L), and chlorophyll-α (µg/L) at a SANAS accredited laboratory (WaterLab Pty) in Pretoria using ICP-MS.

Major and trace metals were measured in water and fish tissues. Water samples were first acidified using 100 μL of ultrapure HNO_3_ (69%), then about 10 mL of water samples was filtered through a 0.45-µm PVDF membrane filter into a 15-mL polyethylene tube using 20-mL disposable sterile syringes. About 0.5 g of fish muscle tissues was treated using 6 mL of aqua-regia for 30 min followed by the addition of 1.0 mL of hydrogen peroxide (30–32% H_2_O_2_) to complete the pretreatment for muscle samples and allowed to react for another 30 min. The fish tissue samples were then digested in a digestion block (DigiBlock Digester ED36S, Labtech, Wilmington, MA). Procedural blanks were prepared in the same procedure as the fish tissue samples. The analysis was done in triplicate. The concentrations of major and trace metals in water and muscle tissue samples were quantified using an iCAP 6500 ICP-OES) (iCAP 6500 Duo series, Thermo Scientific, UK) and an Agilent 7900 ICP-MS (Agilent Technologies Inc., Tokyo, Japan) equipped with an Ultra High Matrix Introduction (UHMI) option, and He cell gas option. The operating parameters of an ICP-OES/ICP-MS were adopted from the manufacturer’s recommendations.

### Water microbial analyses

Sixteen water samples collected from different tanks were serially diluted up to 10-3 and plated on nutrient agar (NA) plates. The plates were incubated at 25 °C for 24 h and distinct colonies were subculture on NA for purification. Based on the different cultural morphologies observed, six different bacterial plates were selected and sent to Inqaba Biotechnology Platform (Pretoria, SA) for identification. Genomic DNA was isolated from the cultures using the Quick-DNA™ Fungal/Bacterial Miniprep Kit (Zymo Research, Catalogue No. D6005). The 16S target region was then amplified using the primers listed below:

- 16S-27 F: AGAGTTTGATCMTGGCTCAG
- 16S-1492R: CGGTTACCTTGTTACGACTT

Cycling conditions included an initial denaturation step at 94 °C for 5 min, 35 cycles of 94 °C for 30 s, 50 °C for 30 s, and 68 °C for 60 s. The final extension was done at 68 °C for 10 min. The integrity of the PCR amplicons was visualized on a 1% agarose (CSL-AG500, Cleaver Scientific Ltd) stained with EZ-vision^®^ Bluelight DNA Dye. The NEB Fast Ladder (N3238) was used as the size standard. The amplicons were purified for sequencing using the Zymo Research, ZR-96 DNA Sequencing Clean-up Kit™, Catalog No. D4050 and sequenced in the forward and reverse direction (Nimagen, BrilliantDye™ Terminator Cycle Sequencing Kit V3.1, BRD3-100/1000) using the ABI 3730*xl* Genetic Analyzer (Applied Biosystems, Thermo Fisher Scientific). The FinchTV (https://finchtv.software.informer.com/1.4/) software was used to view the raw chromatogram files (.abi). CLC Bio Main Workbench was used to assemble the forward and reverse sequencing reads to form a consensus sequence for each sample. BLASTn analysis (with default parameters) (Altschul et al. 1997) was performed on the NCBI website (https://blast.ncbi.nlm.nih.gov/Blast.cgi) to determine if a sequence in the database matches the query sequence above a certain threshold (99% query coverage; 99% identity).

### Statistical analyses

For statistical analyses, the Genstat for Windows 23rd Edition (VSNi, UK) was used. A one-way analysis of variance (ANOVA) and Tukey’s post hoc test were performed to differentiate between means at the 95% probability level. Descriptive statistics included specimen data presented as means and standard deviation and the percentage prevalence of the observed histological alterations. IBM SPSS software was utilized for the analysis of the CF and metals in the control and experimental groups. The Shapiro-Wilk test (*n* < 55) was interpreted to test the data for normality and equal of variance tested using Levene’s test. The non-parametric Mann-Whitey *U* test was utilized to compare the CF of the experimental and control groups. The Mann-Whitney test and independent *T*-test were used for metal concentration analysis comparison.

## Results and discussion

### Water physicochemical properties

The physicochemical water quality parameters in the two tanks were compared with the Department of Water and Forestry (DWAF) (1996) guidelines (Table 1). As shown in Table 1, the mean water quality determinant values were within the DWAF guidelines for agricultural use except for dissolved oxygen, which was lower than the target water quality range, in both tanks. The optimum water temperature for *Oreochromis* growth in an aquaponics system ranges between 22 and 32 °C with a pH level of 5.6–7.3 (Yildiz et al. 2017). The mean temperature was lower in both the control and experimental tanks with the pH falling within the species’ range. The shade net tunnel did not have a temperature regulator which resulted in the low temperatures in the water; however, *O. mossambicus* has been reported to tolerate temperatures as low as 18 °C in aquaculture facilities (James 2014).

**Table 1:** Mean ± SD values of selected in situ water quality parameters recorded during the experimental period

**Parameter**	**Groups**		
**In situ**	**Control tank**	**Experimental tank**	Target water quality ranges (DWAF, 1996)
**Temperature (°C)**	16.3 ± 2.2	19.7 ± 3.9	Vary > 2 °C
**Electrical conductivity (µS/cm)**	405.0 ± 113.4	309.1 ± 59.6	Vary > 15%
**Total dissolved solids (ppm)**	253.5 ± 58.8	178.4 ± 29.8	-
**pH**	6.8 ± 0.3	7.2 ± 0.2	6.0–9.0
**Dissolved oxygen (%)**	73.9 ± 6.8	72.5 ± 1.8	80%–120%

Total dissolved salts and EC were found to be higher in the control group which could be a result of the absence of plants in the system, leading to a high concentration of minerals, organic matter, and salts (Kasozi et al. 2021). Dissolved oxygen was lower than the 80–120% range stipulated by DWAF (1996), and it is important in such a system for the organism to survive and crucial for the oxidation of ammonia (Effendi et al. 2016). Danner (2016) reported that higher EC and TDS increase osmotic stress which may disrupt fish organ function and lead to lower oxygen concentration in the water. According to Zeitoun and Mehana (2014), fish health is negatively affected by poor water quality; however, in this study, the in situ water quality in both tanks was generally conducive for the fish to thrive.

As shown in Table 2, nitrate/nitrite levels were within the DWAF target water quality range before exposure in both control and experimental tanks; however, nitrates were higher than the recommended limit post-harvesting in the control tank. This can be attributed to the absence of potato tubers to absorb these nutrients. Total phosphates were much higher in the control tank and above the DWAF guidelines (DWAF, 1996) but within range for the experimental tanks. This accumulation of phosphates in the control tank can be from the fish waste with no tubers in this system, a high concentration could have resulted.

**Table 2:** Nutrient analysis in sampled water before planting and postharvest

Parameter	Before exposure		After exposure		Target water quality ranges (DWAF, 1996)
	Control tank	Experimental tank	Control tank	Experimental tank	
Nitrate (mg/L)	0.1	0.1	15	1.9	0.5–2.5
Nitrite (mg/L)	< 0.05	< 0.05	< 0.05	< 0.05	0.5–2.5
Total phosphates (mg/L)	0.6	< 0.2	0.6	0.3	0.005–0.250
Free saline ammonia (mg/L)	0.3	< 0.1	< 0.1	< 0.05	0.007
Chlorophyll-α (µg/L)	*	7	*	313	0–30

*Results not available

Free saline ammonia was found to be above the DWAF stipulated guideline before and after exposure in both tanks, which suggests that it was not broken down effectively from the fish waste with the absence of microbes and possibly lower capacity of the biofilters. Chlorophyll-α in the experimental tank, samples were taken after the overnight mortality of twelve fish, was above the DWAF recommended range and this shows a build-up of algae in that system.

The concentrations of Al, Ca, Fe, K, Mg, and Na were measured in water samples collected from the control sump (CS), control tank (CT), experimental sump (ES), and experimental tank (ET), and the results are reported in Table 3. As can be seen, Al and Fe were not frequently detected in water samples, and their concentrations were measured at trace levels. The concentration of Al ranged from 0–35 µg/L, 0–5.83 µg/L, 0–24.2 µg/L, and 0–29 µg/L in the control sump, control tank, experimental sump, and tank, respectively. Iron was not detected in water samples collected from CS, and the Fe concentrations ranged from 0–1.90, 0–500, and 0–1.36 in CT, ES, and ET, respectively. Both elements were higher than the ranges stipulated by DWAF, and elevated aluminium concentrations can impair gill function, thereby reducing oxygen uptake and causing respiratory distress. Moreover, high iron levels may also lead to respiratory distress, increase the susceptibility of fish to infectious diseases, and reduce immune function over time (DWAF 1996).

**Table 3:** Mean ± standard deviation and range of trace elements (µg/L) in water samples from control and experimental tanks and sumps

Sample	Al (µg/L)	Ca (mg/L)	Fe (µg/L)	K (mg/L)	Mg (mg/L)	Na (mg/L)
Control tank	1.06 ± 2.35 (0–5.83)	14.6 ± 6.18 (6.10–24.3)	0.48 ± 0.79 (0–1.90)	6.19 ± 4.23 (1.31–12.5)	5.83 ± 3.05 (1.82–9.58)	16.2 ± 10.0 (3.97–27.8)
Control sump	12.0 ± 17.8 (0–35)	11.5 ± 5.20 (6.26–18.4)	ND	2.96 ± 1.66 (0.9–5.83)	3.62 ± 1.80 (1.12–6.39)	8.28 ± 4.07 (2.37–13.6)
Experimental tank	6.75 ± 11.5 (0–29.0)	17.5 ± 14.7 (5.98–42.1)	0.30 ± 0.55 (0–1.36)	11.4 ± 14.5 (1.39–40.1)	6.81 ± 4.74 (1.69–16.9)	15.3 ± 10.4 (0–24.4)
Experimental sump	9.5 ± 11.0 (0–24.2)	15.5 ± 10.2 (8.82–35.6)	63.0 ± 176 (0–500)	11.2 ± 14.9 (2.40–40.5)	6.16 ± 4.79 (2.75–14.8)	14.8 ± 13.6 (0–44.44)

*ND* not detected

The measured concentrations of Ca, K, Mg, and Na were in sample magnitude in CS and CT. A similar phenomenon was observed for ET and ES. It is worth noting that the ET and ES had higher concentrations compared to CS and CT. Calcium levels were below the TWQR (200–100 mg/L) in both groups and reduced levels of calcium can result in reduced disease resistance making fish prone to many diseases.

The concentrations (range and mean) of As, Cd, Cu, Mn, Pb, and Zn in water samples are presented in Table 4. As shown, the presence of Zn was frequently detected in all the water samples in the concentration range of 17.3–70.2 µg/L, 33.4–588 µg/L, 40.5–289 µg/L, and 0–101 µg/L, respectively. Meanwhile, Mn and Cu were mostly below the detection limits of the instrument.

**Table 4:** Mean ± standard deviation and range of trace heavy metals and metalloids concentration (µg/L) in water samples from the control and experimental tanks and sumps

Sample	As (µg/L)	Cd (µg/L)	Cu (µg/L)	Mn (µg/L)	Pb (µg/L)	Zn (µg/L)
Control tank	0.55 ± 0.93 (0–2.25)	5.2 ± 4.17 (0–9.44)	20.7 ± 27.8 (0–62.1)	3.19 ± 5.11 (0–11.7)	1.56 ± 0.95 (0.56–3.12)	178 ± 216 (33.4–588)
Control sump	2.38 ± 1.41 (1.28–5.10)	5.49 ± 2.97 (0–8.58)	4.78 ± 7.09 (0–18.0)	ND	1.28 ± 0.62 (0.58–2.19)	46.9 ± 19.6 (17.3–70.2)
Experimental tank	3.33 ± 1.99 (0–5.75)	4.35 ± 3.39 (0–8.57)	27.0 ± 46.4 (0–136)	25.3 ± 69.4 (0–197)	1.14 ± 1.12 (0–2.85)	60.3 ± 41.7 (0–101)
Experimental sump	3.93 ± 2.87 (0–6.89)	3.74 ± 3.47 (0–7.26)	30.6 ± 59.7 (0–151)	12.2 ± 29.6 (0–72.7)	1.31± 0.48 (0.43–1.69)	106 ± 94.6 (40.5.29)

*ND* not detected; (), ranges

The presence of toxic heavy metals, As, Cd, and Pb, were detected in more than 80% of the samples at concentrations that are within the DWAF acceptable limits for crop production. It was noted that Mn was detected in the control sump and since there are industrial activities in the study area, the possible source could be from these or commercial feed of fish. In addition, the frequent detection of As and Pb could be due to geogenic sources.

### Fish physiological health

Sodium, Ca, Fe, Mg, and K shown in Table 5 were significantly higher in the experimental group (*p* < 0.05) whereas Al was higher in the control group, but this was not significantly different (*p* > 0.05). Macro- and microminerals such as Na, Ca, K, Mg, and Fe are essential for animals, including fish. As a result, they can be added to fish feed to meet their nutritional requirements.

**Table 5:** Major element concentrations (mg/kg wet wt) in fish muscles in the control and experimental aquaponic tanks presented as mean ± standard deviation

Sample	Ca (mg/L)	Fe (µg/L)	K (mg/L)	Mg (mg/L)	Na (mg/L)
Control (***n*** = 24)	2046.75 ± 5028.59	38.85 ± 48.32	17587.75 ± 17667.77	1193.67 ± 1044.50	1989.04 ± 2140.24
Experimental (***n*** = 13)	4802.23 ± 1989.81	92.55 ± 45.51	39379.23 ± 11147.86	2788.31 ± 399.33	4726.31 ± 1031.05

*n* sample size

The concentrations of As, Cd, Cr, Cu, Mn, Pb, and Zn in fish muscle were investigated, and the mean heavy metal concentrations are summarized in Table 6 which compares their concentrations in fish muscles between the control and experimental tank. The concentration ranges of heavy metals (wet weight basis) in fish muscles from control tanks were 0–1.89, 0–1.30, 0–10.6, 0.97–9.06, 0.11–9.83, 0–3.42, and 6.97–166 mg/kg for As, Cd, Cr, Cu, Mn, Pb, and Zn, respectively (Table 6). The concentration ranges of heavy metals in fish muscles in the experimental tanks were 0–2.30, 0–1.12, 0–29.6, 3.09–187, 1.64–20.8, 0.18–6.25, and 24.1–107 mg/kg for As, Cd, Cr, Cu, Mn, Pb, and Zn, respectively (Table 6).

**Table 6:** Heavy metal concentration (mg/kg wet wt) in fish muscles in the two aquaponics tanks presented as mean ± standard deviation

Sample	As (µg/L)	Cd (µg/L)	Cr (µg/L)	Cu (µg/L)	Mn (µg/L)	Pb (µg/L)	Zn (µg/L)
Control (***n*** = 24)	0.67 ± 0.41	0.73 ± 0.35	3.10 ± 3.29	3.59 ± 2.73	1.79 ± 2.57	1.81 ± 1.30	39.40 ± 41.69
Experimental (***n*** = 13)	1.50 ± 0.47	0.51 ± 0.28	5.64 ± 7.80	20.60 ± 48.11	4.79 ± 5.02	2.87 ± 2.14	59.79 ± 27.78

*n* sample size

On average, fish muscle samples from control tanks had the highest detection frequency of As and Cd. In contrast, Cr and Pb were frequently detected in fish muscle samples from experimental tanks. The elevated concentration of some metals in fish samples from the experimental tanks as compared to those from control tanks suggests that those fish could have accumulated the metal compounds directly from water, sand, and through the feeds. The concentrations of Zn in eight fish out of 24 in the control tank were above the allowed limit of 30 mg/kg set by FAO guidelines (FAO 2022; FAO 1983).

However, only three fish samples from the experimental tanks had Zn concentration within the recommended limits. These results suggest that 16 fish samples from the control tanks and 3 from the experimental tanks were safe from Zn contamination which may have come from the surroundings (feed, water, and sand). The WHO has set the maximum permitted concentration for Cr in fish to 5.0 mg/kg (WHO,^1989^). As seen in Table 6, the Cr content in fish muscles from the control tanks was mostly not detected, and out of the 11 samples where Cr was detected, eight samples had concentrations within the recommended limits. In contrast, Cr was mostly detected in fish samples from experimental tanks, and the concentrations were within the recommended limits, except for two fish samples. Statistical analysis showed Cd and Cr higher in the control group, but this was not significant (*p* > 0.05), and As, Cu, Mn, and Zn were significantly higher in the experimental group (*p* < 0.05).

The maximum permissible limits of Pb and Cd in fish samples are 2 mg/kg and 1 mg/kg as set by FAO (FAO, 1983). Lead was detected in six fish samples from the control tanks, and the mean concentrations of the two samples were above the maximum permissible limit. Lead was detected in all the samples from the experimental tanks at concentrations that mostly exceeded the recommended limit (Table 6). Cadmium was frequently detected in samples from control and experimental tanks at concentrations that were within the accepted limits with few exceptions. Arsenic is a toxic element that has been frequently detected in fish samples and because of its toxicity FAO and WHO have set the permissible limit of this element in fish to 0.05 mg/kg (WHO 1989; FAO 1983).

As shown in Table 6, the concentrations of As were higher than the recommended limit. The recommended limit of Cu in fish is 2.25 mg/kg (FAO/WHO 2011). The Cu concentration in most fish samples from the control and experimental tanks exceeded the permissible limit.

### Fish necropsy

The mean body mass values for the control and experimental groups were 180.72 ± 41.38 g and 108.76 ± 35.27 g, respectively. In terms of sex, the control group had 2% females and 98% males while the experimental group had 46% females and 54% males. There was an overnight mortality of twelve (12) specimens in the experimental tank and one fish in the control group 2 months after planting, and samples were stored for further analysis at a later stage. It should be noted that there was a drastic change in temperature in the shade net tunnel overnight, and due to infrastructure challenges at the time of the experiment, alternative power sources were not available at the center.

**Fig. 2:**
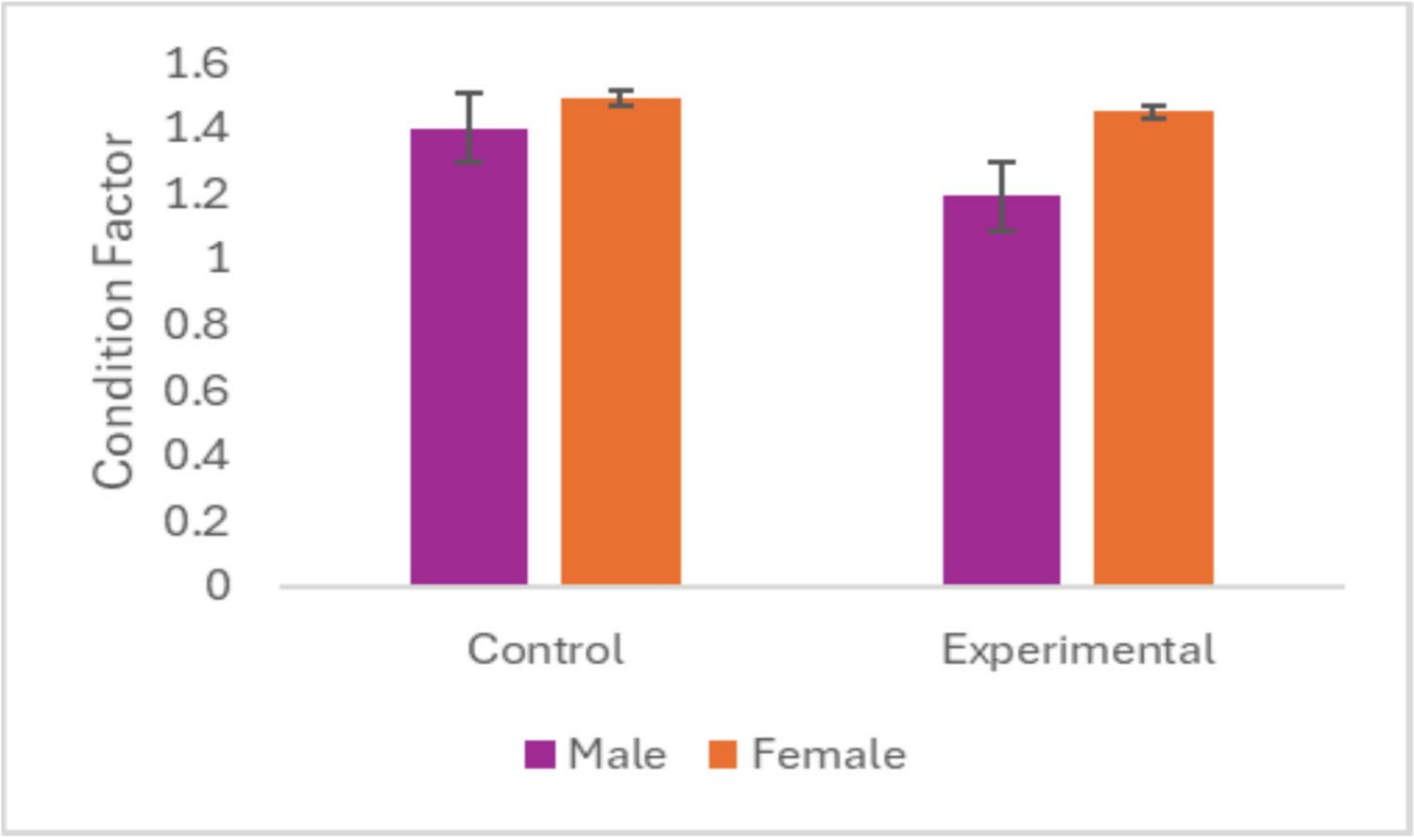
Mean condition factor of *Oreochromis mossambicus* comparing the two groups for males and females

The macroscopic observation of the specimen showed a slightly yellow tint in most of the experimental fish while the control group showed a grayish color. No other abnormalities were observed externally. However, some of the internal organs other than the muscle tissue showed macroscopic abnormalities. The condition factor is a physiological biomarker utilized to assess the general health of fish in biomonitoring studies and can be influenced by food availability and poor water quality (Nibamureke et al. 2019). Results from the current study showed a similar CF (Figure 2) for both groups indicating general good health of the fish as reported by Marchand et al. (2012), and there was no significant difference between the groups differentiated by sex (*p* > 0.05).

### Fish muscle histology

**Fig. 3:**
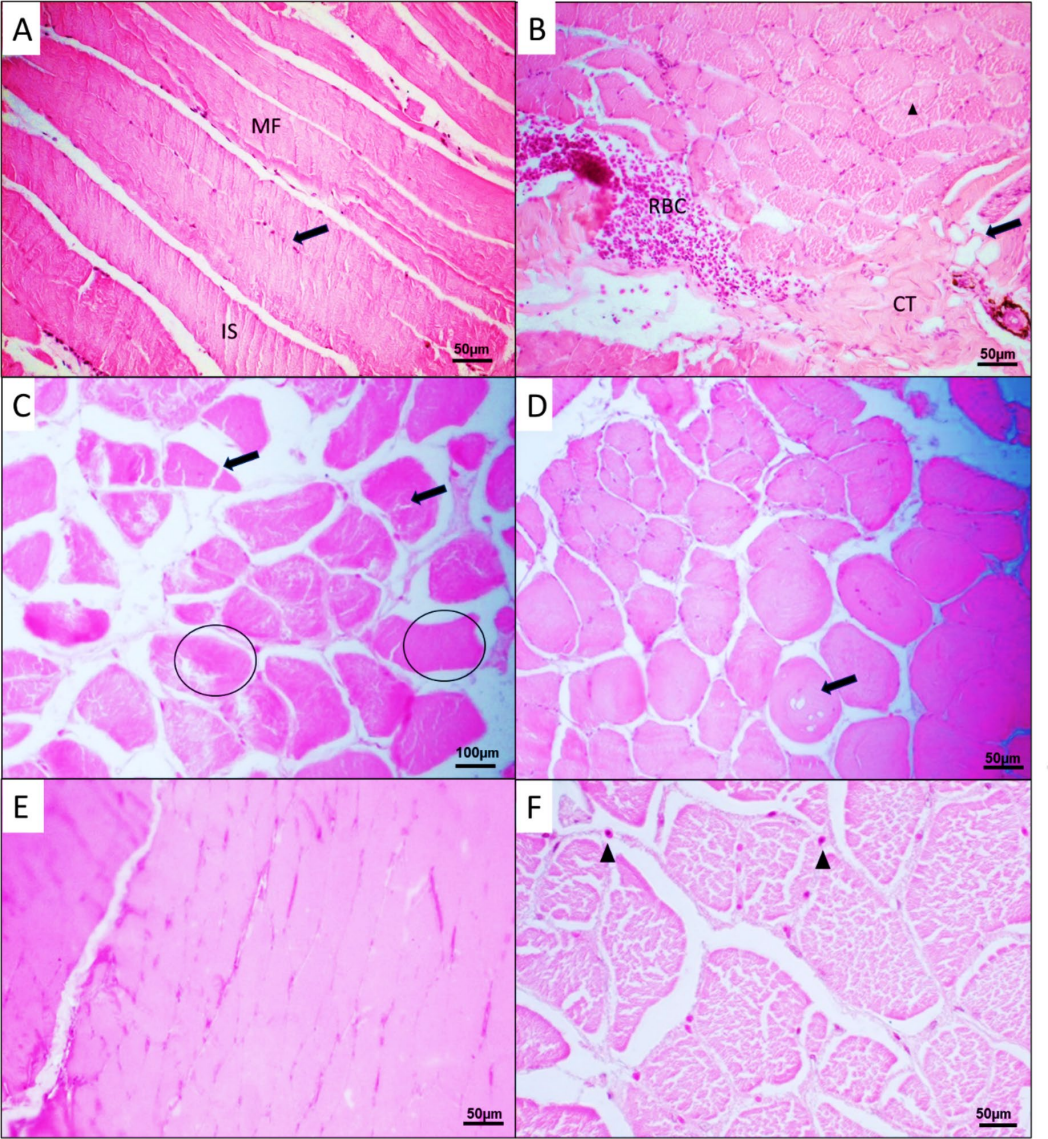
Light micrographs (×10 and ×20) showing the *Oreochromis mossambicus* muscle histological alterations stained with H&E from the control (**A**–**D**) and experimental groups (**E**, **F**). **A** Elongated section of muscle fibers (MF) with nucleus (black arrow) and intracellular spaces (IS). **B** Cross section of the muscle showing connective tissue (CT) with adipocytes (black arrow), muscle fiber splitting (arrowhead), RBC, red blood cells. **C** Muscle fiber splitting (black arrow) and edema. **D** Degeneration of the muscle bundles with cytoplasmic vacuoles. **E** Muscle bundle degeneration. **F** Severe muscle fiber splitting, edema, and nuclei at the periphery. H&E, hematoxylin and eosin

The skeletal muscle of *O. mossambicus* showed elongated muscle fibers arranged in bundles with striations and multiple nuclei at the periphery of the muscle fibers (MF). The epimysium was observed to cover the muscle bundles (MB). Histological alterations were observed in the muscle tissue of both the control and experimental groups (Figure 3). These include the degeneration of muscle bundles, edema, adipocytes in the connective tissue, vacuolation in the muscle fibers, and splitting of the muscle fibers.

There was a higher percentage prevalence of the degeneration of MB, vacuolation, and adipocytes, in the control group whereas edema and splitting of muscle fibers were more prevalent in the experimental group (Table 7). These alterations can be because of the significantly high levels of metals in the fish muscle tissue. According to Jan et al. 2015, metal exposure causes oxidative stress leading to a disruption of tissue structure; however, more alterations were found in the control group. This may suggest that the experimental group had a protective mechanism in place although metals are known to bioaccumulate and bio-magnify. Shahid et al. (2021) stated that edema may be due to infiltration of fluid into the muscle tissue following exposure to heavy metals. These chemicals can lead to the splitting of muscle fibers. Vacuolation was reported by Ullah et al. 2014 in *Cyprinus* *carpio* muscle tissue exposed to contaminated water inundated with heavy metals. Degeneration of muscles was reported by Maharajan et al. (2016) following exposure of *Lates calcarifer* to copper.

**Table 7:** The percentage prevalence of the various histological alterations observed in the muscle tissue of *Oreochromis mossambicus* in both systems

Histological alteration	Percentage prevalence (%)	
	Control (*n* = 24)	Experimental (*n* = 13)
Degeneration in muscle bundles	60	50
Edema	25	40
Adipocytes	60	45
Vacuolation	30	10
Splitting of muscle fibers	40	55

*n* sample size

As the main edible portion of fish, histological alterations in the muscle tissue due to exposure to various metals and poor water quality in an aquaponics system can affect human consumption (Alam et al. 2023). This is because following biomagnification in such a system, metal toxicity can lead to adverse effects on the consumers’ health. Therefore, although SDG 2 is attained, SDG 3 is compromised.

### Water microbial quality

**Fig. 4:**
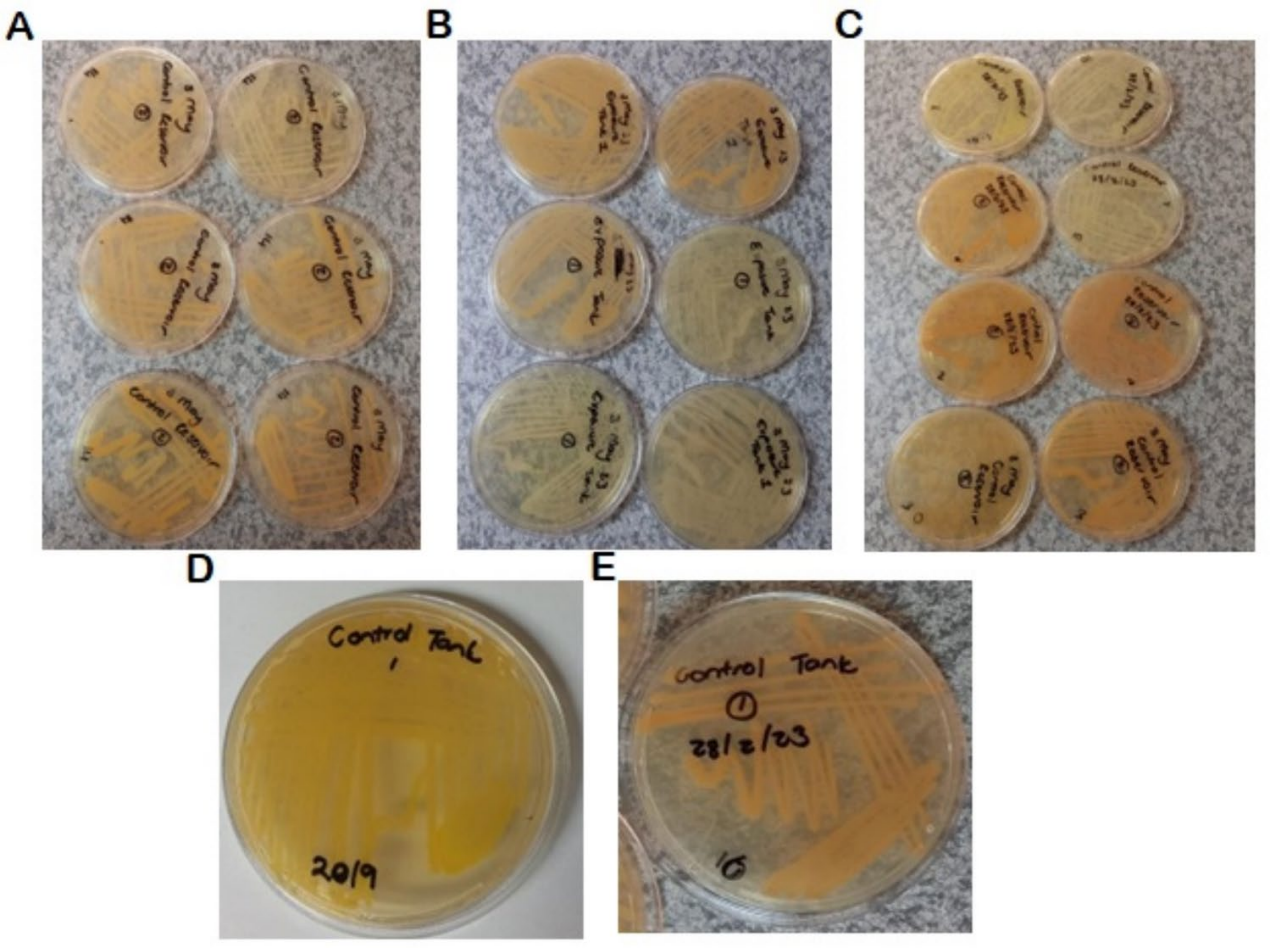
**A** Control sump showing two different colonial morphology. **B** Exposure tank displaying yellow colonies. **C** Control sump tank with two different colonial morphologies. The lemon green was sequenced, and the **D** control tank showed two different colonial morphologies

The following bacterial species were identified from isolates obtained from plated water samples (Figure 4): *Microbacterium oxydans* (99.93% identical), *Exiguobacterium indicum* (99.64% identical), *Exiguobacterium himgiriensis* (99.18% identical), *Pseudomonas putida* (99.86% identical), *Exiguobacterium acetylicum* (99.57% identical), and *Microbacterium oxydans* (99.64% identical) from the control tank (lemon green colonies), control tank 1 (yellow colonies), 3 May control sump (lemon green), 3 May control sump (orange), 3 May 23 exposure tank (yellow colonies), and control sump (lemon green), respectively. Approximately 50% (3/6) of the identified bacteria species belong to the genus *Exiguobacterium*, with 33.3% (2/6) belonging to the genus *Microbacterium* and *Pseudomonas* genus was the least (16.66%; 1/6). *Microbacterium oxydans* is a coryneform Gram-positive bacillus, which has been isolated from terrestrial ecosystems and clinical and food samples. Some *Microbacterium* strains have been shown to produce the plant growth hormone indoleacetic acid, solubilize phosphate, or exhibit 1-aminocyclopropane-1-carboxylate (ACC) deaminase activity (Madhaiyan et al. 2010).

Bacteria of the genus *Exiguobacterium* are Gram-positive facultative anaerobes frequently isolated from various habitats including seawater (Kasana and Pandey 2018), freshwater (Raichand et al. 2012), marine sediment, plant rhizosphere (Khandare et al. 2012), and extreme environments such as lake (Jiang et al. 2013), glaciers, and hot spring (Vishnivetskaya et al. 2011). A study by Hu et al. (2021) revealed that *Exiguobacterium* spp. and *E. acetylicum* were the main spoilage bacteria on fresh-cut leafy vegetables. *Pseudomonas putida* is a Gram-negative bacterium frequently isolated from waters, plants, and soil (Weimer et al. 2020). This bacterium has been reported to infect tomato plants in Italy (Dimartino et al. 2011). Eck et al. (2019) found that Proteobacteria and Bacteroidetes were the most predominant in aquaponic systems using *O. niloticus*.

### Potato yield performance analyses

The results on tuber quality revealed that Moonlight produced the heaviest tubers out of the two cultivars used in the study. Table 8 shows this to have been consistent in the two grow beds used to produce potatoes. The number of tubers per plant was similar between the two cultivars across both beds. The Tuber Form Index indicates Moonlight produced more elongated tubers (short oval), while the Taurus tubers produced were more round or slightly oval depending on the bed. This indicates hydroponic system produced Moonlight may be more suitable for applications where larger, more elongated tubers are desired, such as French fry production, while Taurus may be better suited for fresh market or table use where rounder tubers are preferred (Ezekiel et al. 2013).

**Table 8:** Yield components (per plant) and shape of harvested tubers

Cultivar	Weight (g)	Number of tubers	TFI	Shape
Taurus Bed 1	168 ± 57	6 ± 3	107 ± 15	Round
Moonlight Bed 1	307 ± 198	7 ± 4	129 ± 12	Short oval
Taurus Bed 2	139 ± 51	6 ± 4	114 ± 21	Short oval
Moonlight Bed 2	293 ± 152	8 ± 4	118 ± 9	Short oval

The average tuber weight for the Moonlight was 293–307 g while that of Taurus was 139–168 g. The difference was expected as tuber weight in potatoes can vary greatly depending on the cultivar (Vos 1999). However, tubers produced in this study were comparable to those produced under field conditions. Potato cultivars grown under optimal field conditions can range from 100 to 300 g per tuber, as observed in studies on commercial varieties such as Russet Burbank and Desiree. Moonlight produced in this study fits into the higher end of the weight spectrum, while Taurus falls on the lower end, which suggests that Moonlight has a stronger yield potential under a hydroponic system. Notwithstanding, the number of tubers per plant in both cultivars (6–8 tubers) was also within the common range reported in other research. Typical potato plants produce between 6 and 12 tubers per plant depending on the cultivar, planting density, and environmental factors. Studies have shown that tuber numbers tend to decrease as individual tuber weight increases due to a trade-off between the two factors. This was consistent in the current study, as the heavier tubers of Moonlight had slightly fewer tubers than the lighter-yielding cultivar, Taurus.

The nutritional profile of the Taurus and Moonlight potato cultivars grown in two different beds revealed several key differences and similarities (Table 9). Moisture content was similar across all potato samples, ranging from 12 to 13%. Taurus from Bed 1 had the highest total fiber content (16%) and neutral detergent fiber (NDF) content (11%), while Moonlight from both beds had lower fiber levels (11–12%). Protein content varied, with Taurus in Bed 2 having the highest at 10.4% and Moonlight in Bed 1 the lowest at 9.6%. Both cultivars showed similar ash and potassium (K) content, though Taurus in Bed 2 had slightly higher magnesium (Mg) and iron (Fe) levels. Phosphorus (P), zinc (Zn), manganese (Mn), and copper (Cu) were comparable across all samples, with minor variations.

**Table 9:** Nutritional profile of harvested tubers

Cultivar	Moisture (%)	Total fiber (%)	NDF (%)	ADF (%)	Protein (%)	Ash (%)	K (%)	Ca (%)	Fe (ppm)	Mg (%)	P (%)	Zn (ppm)	Mn (ppm)	Cu (ppm)
Taurus Bed 1	13 ± 0.9	16 ± 5.4	11 ± 4	6 ± 1.5	8.8 ± 2	4.6 ± 0.7	1.3 ± 0.1	0.018 ± 0.005	111 ± 32	0.098 ± 0.005	0.0925 ± 0.010	12.0 ± 3.92	7 ± 0.00	2.0 ± 0.82
Moonlight Bed 1	12 ± 0.4	12 ± 4.9	7 ± 3	4 ± 1.5	9.6 ± 0	4.7 ± 1.2	1.3 ± 0.2	0.018 ± 0.008	104 ± 33	0.096 ± 0.015	0.084 ± 0.015	8.8 ± 9.58	7.8 ± 2.28	2.6 ± 0.55
Taurus Bed 2	13 ± 0.7	11 ± 2.8	7 ± 2	4 ± 0.5	10.4 ± 1	4.4 ± 1.2	1.4 ± 0.1	0.022 ± 0.008	143 ± 25	0.104 ± 0.005	0.092 ± 0.013	16.0 ± 4.36	8.2 ± 1.10	2.6 ± 0.55
Moonlight Bed 2	12 ± 0.4	11 ± 1.2	7 ± 0	4 ± 0.8	9.8 ± 0	3.8 ± 0.8	1.3 ± 0.1	0.015 ± 0.006	79 ± 34	0.098 ± 0.005	0.085 ± 0.006	4.0 ± 1.73	7 ± 0.00	2.5 ± 0.58

*NDF* neutral detergent fiber, *ADF* acid detergent fiber

When comparing the nutritional profile of potato cultivars in this study to findings in the literature, several notable trends can be observed. The total fiber content in samples (11–16%) was significantly higher than what is generally reported in the literature. Potatoes are commonly noted to contain around 2.0–3.3% dietary fiber. The increased fiber could be due to specific cultivar traits. The protein content in tubers (8.8–10.4%) was higher than the average protein levels for most potato varieties, which range between 1.5 and 2.5%. This may reflect a higher-protein cultivar or specific environmental conditions that promote protein synthesis in the tubers (Dwelle 2014). With regard to mineral content, potassium (K) levels in samples (1.3–1.4%) align with typical values reported for potatoes, which generally range from 1.1 to 2.7%.

Iron (Fe) levels (79–143 ppm) are within the usual range of 60–160 ppm in potatoes. Magnesium (Mg) and phosphorus (P) contents are in line with existing literature, with Mg generally reported around 0.1–0.2% and P levels at 0.07–0.15%. The ash content in samples was 3.8–4.7%, which is higher than typical literature values, which range between 2 and 3%. Ash content reflects the total mineral content and could indicate a higher mineral density in these cultivars, which could be explained by the relatively small tuber size produced in this experiment.

## Conclusion

Making aquaponic systems sustainable involves finding the right balance for the environment in which three different organisms thrive. It is about maximizing what we produce while minimizing any waste that might harm the environment. Potato quality showed a higher than usual fiber and protein content compared to average potato values reported in the literature, which likely reflects specific environmental influences. These differences could also reflect the cultivar traits of Taurus and Moonlight which interact with environmental factors, such as nutrient availability. The biometric index (CF) showed that fish from both systems were generally in good health, although some metals were significantly higher in the fish muscle tissues from the control group and there was a higher percentage prevalence of histological alterations. This study emphasizes the intricate relationship between plants, water quality parameters, and fish health in sandponics systems. The presence of potato tubers had a notable impact on water quality and fish health, with the experimental group exhibiting improved overall water quality despite higher levels of heavy metals. Although the system is sometimes beneficial for lowering the costs of crop production, drawbacks have been cited regarding its elevated maintenance requirements, which lead to low-profit margins (Lobillo-Eguíbar et al. 2020). Given its potential to sustain yields comparable to conventional production, further research is warranted to investigate the physiological growth performance of crops and to identify opportunities for system optimization. In addition, the assessment of other target organs (i.e., the liver) of *O. mossambicus* can provide valuable insights into the edibility of fish in such a system. One of the main challenges is that aquaponic systems require constant and careful maintenance.

## Statement

During the preparation of this work, the author(s) used ChatGP3.0 (generative AI) and PaperPal to improve the readability and language. After using this tool, the author(s) reviewed and edited the content as needed and took full responsibility for the content of the publication.

## Data Availability

Data is available upon request from all co-authors.
